# Age and sex distribution of beat-to-beat blood pressure variability after transient ischemic attack and minor stroke: A population-based study

**DOI:** 10.1177/1747493020971905

**Published:** 2020-11-09

**Authors:** Alastair JS Webb, Amy Lawson, Sara Mazzucco, Linxin Li, Peter M Rothwell

**Affiliations:** Wolfson Centre for Prevention of Stroke and Dementia, Department of Clinical Neurosciences, John Radcliffe Hospital, University of Oxford, UK

**Keywords:** Age, sex, blood pressure variability, transient ischemic stroke, TIA, minor stroke, hypertension

## Abstract

**Background:**

Beat-to-beat blood pressure variability is associated with increased stroke risk but its importance at different ages is unclear.

**Aims:**

To determine the age-sex distribution of blood pressure variability in patients with transient ischemic stroke or minor stroke.

**Methods:**

In consecutive patients within six weeks of transient ischemic stroke or non-disabling stroke (Oxford Vascular Study), non-invasive blood pressure was measured beat-to-beat over five minutes (Finometer). The age-sex distribution of blood pressure variability (residual coefficient of variation) was determined for systolic blood pressure and diastolic blood pressure. The risk of top-decile blood pressure variability was estimated (logistic regression), unadjusted, and adjusted for age, sex, and cardiovascular risk factors.

**Results:**

In 908 of 1013 patients, excluding 54 in atrial fibrillation and 51 with low quality recordings, residual coefficient of variation was positively skewed with a median systolic residual coefficient of variation of 4.2% (IQR 3.2–5.5) and diastolic residual coefficient of variation of 3.9% (3.0–5.5), with 90th centile thresholds of 7.2 and 7.3%. Median systolic residual coefficient of variation was higher in patients under 50 years (4.5 and 3.0–5.3) compared to 60–70 years (4.1 and 3.2–5.2), but rose to 4.5% (3.5–6.9) above 80 years, with an increasingly positive skew. The proportion of patients with markedly elevated blood pressure variability in the top-decile increased significantly per decade (OR 1.72, p < 0.001), after adjustment for sex and risk factors.

**Conclusions:**

Median beat-to-beat blood pressure variability fell in midlife, reflecting loss of physiological, organized blood pressure variability. However, rates of markedly elevated blood pressure variability significantly increased with greater age, suggesting that blood pressure variability may be particularly important in older patients.

## Introduction

Patients with episodic hypertension have a high risk of stroke,^1,2^ residual visit-to-visit variability in blood pressure (BPV) on treatment has a poor prognosis despite good control of mean blood pressure (BP),^3^ and benefits of some antihypertensive drugs in the prevention of stroke may partly result from reduced variability in systolic blood pressure (SBP).^4,5^ Strong associations for visit-to-visit BP variability have been found with recurrent cardiovascular events,^6^ diabetes,^7^ renal impairment,^8^ and cognitive decline,^9^ with similar predictive value of BP variability on day-to-day home readings^10,11^ and a significant reduction with specific antihypertensives.^4,5^ However, both visit-to-visit and home BP variability require a prolonged period of assessment, good patient compliance, and follow-up visits. As such, they are of limited use in acute assessment.

Variability in BP from one beat to the next (beat-to-beat BPV) enables BPV assessment at a single visit and is associated with an increased risk of recurrent stroke and cardiovascular events in patients with a transient ischemic attack (TIA) or minor stroke, with a similar physiological profile to home day-to-day BPV^12^ and at least similar predictive value.^11^ However, normal values for beat-to-beat BPV in at-risk individuals and their distribution across age and sex groups are unknown. Furthermore, beat-to-beat BPV is itself composed of multiple components from physiological rhythms reflecting breathing and intact autonomic function to increased BPV associated with stiff arteries and impaired baroreceptor function in older patients with impaired compensatory mechanisms.^12^ To assess the potential clinical utility of beat-to-beat BPV, it is necessary to understand the distribution of beat-to-beat BPV in at-risk populations and whether potentially pathological, markedly increased BPV is increased in specific groups.

Therefore, we determined the normative age-sex distribution of beat-to-beat BPV variability in patients with a TIA or minor stroke.

## Methods

### Study population

Consecutive, consenting patients with TIA or minor stroke were recruited between September 2010 and October 2019 to the Oxford Vascular Study (OXVASC) Phenotyped Cohort.^11,12^ Participants were recruited at the daily emergency clinic, following a referral after attendance at the Emergency Department or from primary care, usually within 24 h. The OXVASC population consists of >92,000 individuals registered with 100 primary-care physicians in Oxfordshire. All consenting patients underwent a standardized medical history and examination, ECG, blood tests, and a stroke protocol MRI brain and contrast-enhanced MRA (or CT-brain and carotid Doppler ultrasound or CT-angiogram). All patients were assessed by a study physician and reviewed by the senior study neurologist (PMR) and are followed-up face-to-face at 1, 3, 6, and 12 months and 2, 5, and 10 years. Access to the data will be openly considered on application to the chief investigator (PMR).

As part of the OXVASC Phenotyped Cohort, a routine prospective cardiovascular physiological assessment is performed at the one-month follow-up visit. Participants were excluded if they were under 18 years, had severe cognitive impairment, were pregnant, had autonomic failure, or had a recent myocardial infarction, unstable angina, heart failure (NYHA 3–4 or ejection fraction <40%), or untreated bilateral carotid stenosis (>70%). OXVASC is approved by the Oxfordshire Research Ethics Committee.

After 15–20 min supine rest, beat-to-beat BPV was measured over five minutes in a quiet, dimly lit, temperature-controlled room (21–23℃). Continuous ECG and non-invasive BP were acquired at 200 Hz (Finometer, Finapres Medical Systems, The Netherlands) via a Powerlab 8/30 (LabChart Pro, ADInstruments, USA). Waveforms were preferentially recorded from the middle phalanx of the middle finger. Automated calibration (“Physiocal”) was performed until the recording was stable, but turned off during each test. Estimated brachial waveforms (Finometer) were calibrated offline by linear regression to 2–3 supine, oscillometric brachial readings, performed immediately prior to the monitoring period on the contralateral arm, with manual exclusion of artifacts. Measurements were not adjusted for differences in BP between arms. If necessary, the finger cuff was moved to an adjacent finger or the proximal phalanx of the same finger or the hand was warmed. Two sitting clinic BPs, five-minutes apart, were measured at ascertainment and one month in the non-dominant arm by trained personnel.

### Analysis

BPV on beat-to-beat monitoring was calculated over five minutes. Ectopic beats and artifacts were automatically detected, visually reviewed, and removed by linear interpolation of R–R interval. BP artifacts were automatically detected and manually reviewed and removed by linear interpolation to adjacent normal beats, with in-house software. Patients in atrial fibrillation during the recording were excluded. Systolic and diastolic BPV were calculated as the standard deviation (SD) and the coefficient of variation (CV = SD/mean), before and after detrending of the recording about a linear regression (rCV). All recordings were reviewed blinded to clinical data to assess for the quality of recording (3—excellent quality; 2—adequate quality for analysis; 3—unusable, poor quality recording).

Distributions of BP indices were described by histograms for all patients, stratified by sex and age. Normality of distributions was assessed by the Shapiro–Wilk test. Summary values for non-normal distributions (p < 0.05) were summarized as the median, skewness, inter-quartile range, and 90th centile.

Models were performed for univariate associations; adjusted for age and sex and for age, sex, and cardiovascular risk factors (current smoking, history of hypertension, and diabetes).

## Results

1031 Assessments were performed in 1013 eligible, consecutive, consenting patients between September 2010 and October 2019, with 18 patients assessed twice. Of 1013 patients, 54 (5.3%) were in atrial fibrillation during the recording, whilst 51 (5%) patients had inadequate recordings. Patients with atrial fibrillation or poor recording quality were older, had higher BP, and were more likely to have a history of hypertension (Table 1).

**Table 1.** Demographics of participants.

	All	Included	In AF	Poor data	p-val
	1013	908 (90)	54 (5)	51 (5)	
Age^a^	67 (13.3)	66.1 (13.3)	77.8 (8.9)	72.2 (11.1)	<0.001
Sex	427 (42)	387 (43)	18 (33)	22 (44.9)	0.35
Hypertension	453 (44)	390 (43)	36 (67)	27 (55.1)	<0.001
Diabetes	116 (11)	104 (11.5)	7 (13)	5 (10.2)	0.88
Smoking					
Current	160 (15)	148 (16)	6 (11)	6 (12.2)	0.39
Ever	535 (53)	478 (53)	32 (59)	24 (49)	0.62
Family history	303 (30)	274 (30)	12 (22)	17 (34.7)	0.12
AF					
During test	54 (5.3)	–	54 (100)	–	–
Ever	74 (7)	37 (4.1)	35 (65)	2 (4.1)	<0.001
Dyslipidemia	314 (31)	277 (31)	20 (37)	16 (32.7)	0.67
Heart failure	16 (1.6)	10 (1.1)	6 (11)	–	<0.001
Systolic BP^a^	133 (19)	132 (18)	137 (24)	145 (27)	<0.001
Diastolic BP^a^	78 (12)	78 (11)	83 (15)	77 (8.9)	0.01
Antihypertensives					
ACEI	438 (43)	394 (43)	27 (50)	17 (33)	
ARB	133 (13)	111 (12)	11 (20)	11 (22)	
CCB	458 (45)	403 (44)	25 (46)	30 (59)	
Diuretic	325 (32)	288 (32)	23 (42)	14 (27)	

AF: atrial fibrillation; BP: blood pressure' ACEI: angiotensin converting enzyme inhibitor; ARB: angiotensin receptor blocker; CCB: calcium channel blocker. Data are shown as frequency (%) unless otherwise specified. Data are shown as frequency (%) unless otherwise specified. ^a^data are shown as mean (standard deviation)

Mean SBP was strongly correlated with SD of SBP (Supplementary Figure 1) but with no correlation with CV–SBP or CV–DBP, before or after detrending. However, there was correlation between mean–DBP and SD–DBP, with an inverse correlation between CV–DBP and mean DBP before and after detrending (Supplementary Figure 1).

**Figure 1.**
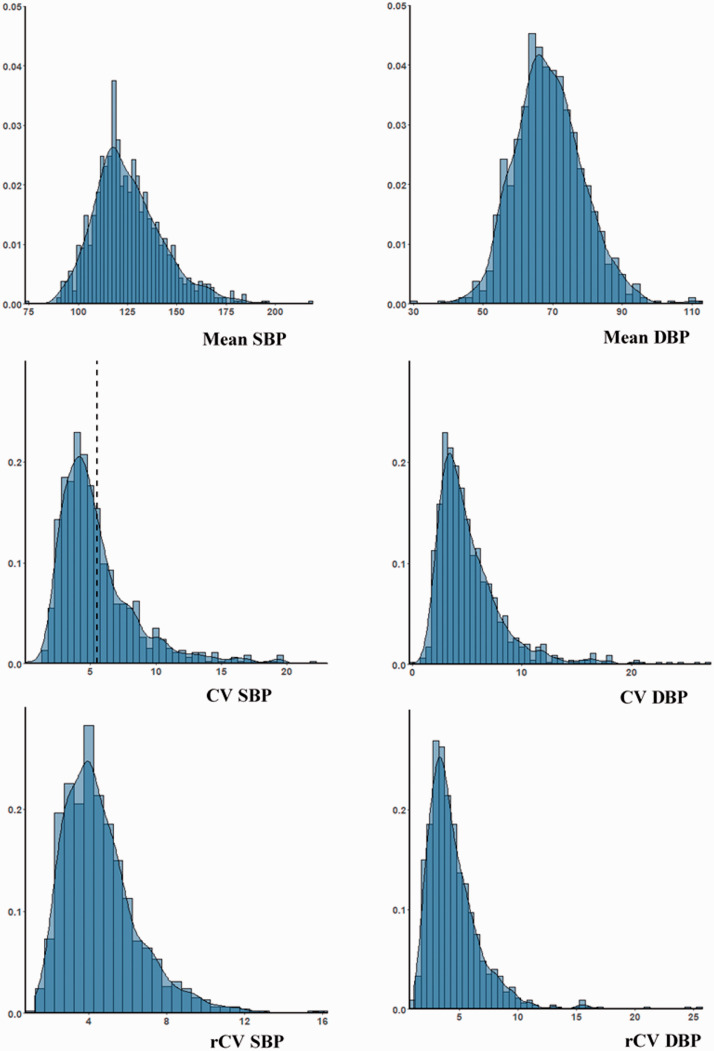
Distribution of mean and variability in systolic and diastolic blood pressure across the population. Histograms are presented with density curves. BP variability is presented as coefficient of variation (CV) and as residual coefficient of variation after linear detrending of the data (rCV, %). SBP: systolic blood pressure; DBP: diastolic blood pressure.

Mean SBP and DBP were normally distributed across the population, but all indices of BPV were positively skewed (Figure 1), with a similar skew for maximum BP values (Supplementary Figure 2) and a greater skew for DBP than SBP (Figure 1). There was a persistent but reduced skew after detrending of recordings (Table 2, Supplementary Table 2). Median rCV–SBP was greater than median rCV–DBP (Table 1), but the distribution of rCV–DBP was more skewed, with a higher threshold for the top decile of the population. Although mean DBP was greater in men than women (Supplementary Figure 1), there was minimal difference in the distribution of either CV (Supplementary Figure 2) or rCV (Supplementary Figure 3).

**Table 2.** Age-dependent distribution of residual coefficient of variation (rCV) for systolic and diastolic blood pressure

	*n*	rCV–SBP				rCV–DBP			
		Median (IQR)^a^	90%^b^	p-val	Skew^c^	Median (IQR)^a^	90%^b^	p-val	Skew^c^
All	908	4.2 (3.2–5.5)	7.18	<0.001	1.4	3.9 (3.0–5.5)	7.34	<0.001	2.8
Age									
<50	110	4.5 (3.0–5.3)	6.17	0.006	0.5	4.3 (3.2–5.7)	6.82	<0.001	3.0
50–60	162	4.0 (3.4–5.3)	6.97	<0.001	1.2	3.7 (2.9–4.9)	7.46	<0.001	1.8
60–70	222	4.1 (3.2–5.2)	6.72	<0.001	1.3	3.7 (2.8–5.1)	6.69	<0.001	3.5
70–80	293	4.4 (3.1–5.6)	7.36	<0.001	1.4	4.0 (3.0–5.5)	6.85	<0.001	1.6
>80	121	4.5 (3.5–6.9)	8.47	<0.001	1.2	4.7 (3.3–6.6)	9.72	<0.001	2.2

a Results the median value, inter-quartile range (IQR). ^b^90th centile. ^c^skewness. p-Values for normality (Shapiro Wilk).

**Table 3.** Demographic associations with being in the top decile of blood pressure or blood pressure variability

	Systolic BP				Diastolic BP			
	Mean		rCV		Mean		rCV	
	OR^a^	p-val	OR^a^	p-val	OR^a^	p-val	OR^a^	p-val
Unadjusted								
Age	1.68	<0.001	1.57	<0.001	0.83	0.019	1.08	0.37
Female	1.08	0.744	1.58	0.044	0.53	0.008	1.20	0.429
Adjusted (A/S)								
Age	1.64	<0.001	1.55	<0.001	0.86	0.061	1.06	0.454
Female	1.01	0.978	1.50	0.079	0.55	0.011	1.18	0.461
Adjusted (CVrf)								
Age	1.64	<0.001	1.72	<0.001	0.84	0.066	1.09	0.36
Female	1.03	0.902	1.42	0.142	0.53	0.011	1.09	0.724

a Odds ratios (OR) are presented per decade with p-values (p-val) from logistic regression, unadjusted, and adjusted for age/sex, or age, sex, and cardiovascular risk factors.

rCV: residual coefficient of variation after linear detrending.

The median value of rCV–SBP and rCV–DBP increased across age groups. However, there was a greater median rCV in patients under 50 compared to patients aged between 50 and 60, although the distribution of CV was more “normal.” However, rCV was more positively skewed with increasing age, with a progressive increase in the top decile of rCV across groups, particularly in patients over the age of 80, suggestive of a non-physiological increase in BPV.

**Figure 2.**
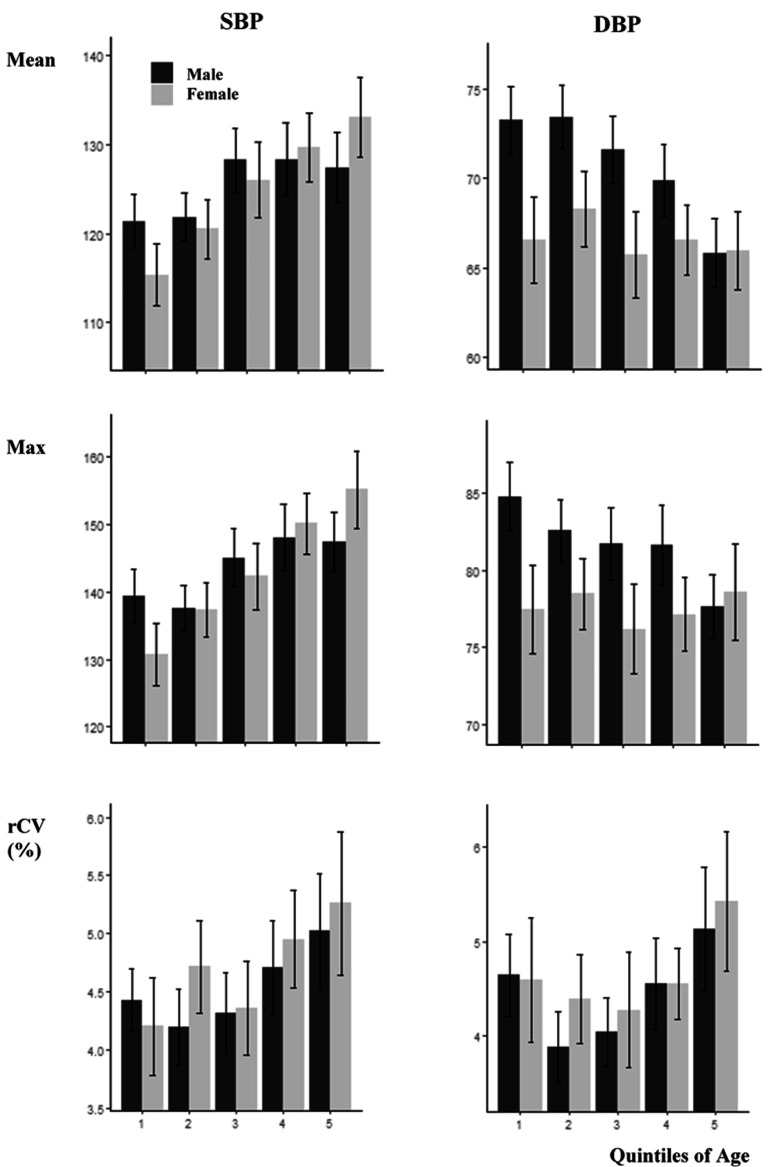
Mean values of mean, maximum and rCV for systolic (SBP) and diastolic (DBP) blood pressure, stratified by sex and quintiles of age. Results are presented as the mean and confidence interval for each group. rCV is calculated as the coefficient of variation of de-trended recordings. Age groups are given in quintiles: <54.2; 54.3–64.7; 64.8–71.4; 71.5–77.7; >77.7 years.

In contrast to the greater linear increase in mean SBP with age in women than men, the non-linear change in rCV was similar for both men and women but was more marked for DBP, with a nadir of rCV–DBP occurring at a greater age in women than men (Figure 2). However, there was minimal difference in the median rCV–DBP in hypertensive patients, diabetics, and smokers, with only a slightly greater CV–SBP in smokers and diabetics (Table 2).

The risk of being in the top decile was associated with increased age and sex in univariate analyses for rCV–SBP but not for rCV–DBP, with a similar pattern for mean SBP and an inverse association for mean DBP (Table 3). After adjustment for age, the association with sex was not significant. The same pattern of association was seen for general linear models for the association between demographics measures and rCV (Supplementary Table 3).

## Discussion

In this high-risk population, beat-to-beat BP variability over five minutes was positively skewed with a median rCV of 4.2% for systolic BP and 3.9% for diastolic BP. However, there was a non-linear relationship between rCV and age with a fall in BPV in younger patients, likely reflecting loss of intact physiological mechanisms, followed by a high rate of markedly increased BPV in older age-groups, implying that these patients may be at particular risk from excess BPV.

Despite studies demonstrating that visit-to-visit and day-to-day BP variability is associated with an increased risk of cardiovascular events,^1–4,7,9,13–15^ few studies have determined the prognostic significance of beat-to-beat blood variability^11,16^ despite its widespread use in the assessment of autonomic function.^17^ We previously demonstrated that beat-to-beat BP variability was associated with a 47% increased risk of stroke and 37% increased risk of cardiovascular events per standard deviation increase in beat-to-beat BPV^11^ compared to 24 and 33% for day-to-day BP variability. One other study demonstrated that beat-to-beat BPV was increased in acute stroke and associated with poor outcome,^16^ albeit with SD as the principle index of BPV. Beat-to-beat BPV was also associated with markers of end-organ injury in this population,^12^ in limited studies in other populations,^10,18^ and in limited studies using intra-arterial BP.^19^ This is consistent with the similar associations of beat-to-beat, visit-to-visit, and day-to-day BPV with physiological mechanisms,^12^ the similar prognostic value of beat-to-beat and day-to-day BPV in this population,^11^ and their similarity to the prognostic value of visit-to-visit BPV.^2^ However, beat-to-beat variability reflects short-term physiological processes, such as those driven by the autonomic nervous system, and does not reflect intra-individual BPV due to longer-term factors such as time of day, day-to-day influences such as weekday versus weekend or seasonal factors, which will only impact upon inter-individual differences in beat-to-beat BPV. As such, its prognostic significance may be driven by different underlying processes and have different relevance in different patient groups.

There is still no agreement as to the optimal measure of BPV or a description of its distribution in at-risk populations. In this study, we confirmed that SD was strongly correlated with mean BP, whilst CV–SBP was not significantly associated with mean–SBP despite a weak negative correlation between mean DBP and CV–DBP. However, due to the baseline drift in BP during recordings, due either to measurement artefact or a physiological reduction in baseline BP, detrending about a linear regression significantly reduced the artifactual component of the positive skew of the population. Although previous studies have also derived variation independent of the mean,^1,3^ this is a population-specific measurement that cannot be applied in an individual or be used to derive normative values.

BPV was very positively skewed, and this increased with age. As such, dichotomized thresholds for markedly elevated BPV are likely to have prognostic significant. Given an increased risk of recurrent events in the top quartile of BPV in this population,^11^ with a particularly high risk in the top decile of BPV in analyses of visit-to-visit BPV,^2^ 75% and 90% population thresholds for rCV–SBP are likely to be clinically applicable. Furthermore, the association between age and beat-to-beat BPV was markedly non-linear, consistent with reports from the ASCOT–BPLA and UK–TIA trials,^1,2^ with similar associations between age and other outcomes.^20,21^ As such, simple linear adjustment for age in epidemiological models of BPV and similar variables, as is the norm, will consistently fail to adequately adjust for age in prognostic models, resulting in systematic bias. Either stratified models or non-linear approaches^20,22^ are therefore essential to develop age-specific prognostic estimates for key predictors of future cardiovascular risk.

Despite the positive skew of the distribution of BPV, BPV was greater in the youngest patients compared to patients over 50 years old, with a more normal distribution. However, as age increased, there was a progressively greater skew of the population with an increasing threshold for the top decile of rCV–SBP across age strata. This likely reflects different components of BPV, with a fall in physiological, ordered fluctuations in younger patients reflecting an intact autonomic nervous system, followed by later-life development of increased, more randomly distributed BPV in patients with failure of compensatory mechanisms.

There are limitations to our study. First, all patients were assessed for a cerebrovascular event, limiting generalizability to other groups. However, this population is at an increased risk of recurrent stroke^23^ associated with increased beat-to-beat BPV.^11^ Second, 5% of patients did not have adequate recordings despite methods to improve quality, particularly in elderly patients who may be at a particularly increased risk of stroke. As such, the prevalence of elevated BPV and associated cardiovascular risks may be underestimated. Third, we measured beat-to-beat BPV in a highly controlled environment, using expensive equipment (Finometer). Development of more cost-effective methods would be essential to apply beat-to-beat BPV to routine clinical practice. Fourth, patients with atrial fibrillation were excluded from the analysis due to confounding by the randomly distributed variation in the R–R interval and the increased stroke risk due to embolism, while the technique used has not been validated in patients with AF. Additional research is required to validate or develop measurement techniques in AF although more patients will be required to investigate the determinants and significance of beat-to-beat BPV in patients with AF. Finally, we extensively cleaned and de-trended the data, improving precision of measurement but also limiting its applicability to clinical practice. As such, further development is required to standardize methods of acquisition, data cleaning, and analysis of beat-to-beat BPV in a cost-effective and practical method.

Overall, we determined the distribution of beat-to-beat BPV in a relevant, high-risk population with TIA or minor stroke, providing normative values and characterization of the distribution by age and demographic group. This lays the foundation for further research to characterize the physiological determinants of BPV and to differentiate BPV reflecting intact, protective physiological processes from abnormal BPV associated with an increased risk of stroke. Furthermore, this study will enable us to investigate the difference in BPV between different stroke etiologies and vascular anatomy, including the effect of vascular stenosis proximal to the site of measurement. This will allow further analysis of the prognostic significance of beat-to-beat BPV in large populations and trials to determine its validity as a target for treatment in specific patient groups. Finally, it will be critical to develop practical methods to measure BPV in a cost-effective manner in clinical environments. More user-friendly devices have recently become available, but their prognostic significance and utility in measuring BPV need to be validated.

Beat-to-beat BP variability is a promising method to characterize BP variability at the time of a single patient assessment. We have characterized the distribution of BPV in a high-risk population, identifying a high rate of markedly elevated BP in older patients. As well as providing evidence of different forms of BPV, this provides a robust foundation for future studies to better characterize the prognostic significance of beat-to-beat BPV and its utility as a treatment target.
